# Aberrant expression and constitutive activation of STAT3 in cervical carcinogenesis: implications in high-risk human papillomavirus infection

**DOI:** 10.1186/1476-4598-9-282

**Published:** 2010-10-27

**Authors:** Shirish Shukla, Gauri Shishodia, Sutapa Mahata, Suresh Hedau, Arvind Pandey, Suresh Bhambhani, Swaraj Batra, Seemi F Basir, Bhudev C Das, Alok C Bharti

**Affiliations:** 1 Division of Molecular Oncology, Institute of Cytology & Preventive Oncology, I-7, Sector-39, NOIDA, U.P.-201301, India; 2 Division of Cytopathology, Institute of Cytology & Preventive Oncology, I-7, Sector-39, NOIDA, U.P.-201301, India; 3 Department of Gynae and Obstetrics, Lok Nayak Hospital, New Delhi, 110002, India; 4 Department of Biosciences, Jamia Millia Islamia, New Delhi, 110025, India; 5 Dr. B.R. Ambedakar Centre for Biomedical Research, University of Delhi, New Delhi, 110029, India

## Abstract

**Background:**

Recent observations indicate potential role of transcription factor STAT3 in cervical cancer development but its role specifically with respect to HPV infection is not known. Present study has been designed to investigate expression and activation of STAT3 in cervical precancer and cancer in relation to HPV infection during cervical carcinogenesis. Established cervical cancer cell lines and prospectively-collected cervical precancer and cancer tissues were analyzed for the HPV positivity and evaluated for STAT3 expression and its phosphorylation by immunoblotting and immunohistochemistry whereas STAT3-specific DNA binding activity was examined by gel-shift assays.

**Results:**

Analysis of 120 tissues from cervical precancer and cancer lesions or from normal cervix revealed differentially high levels of constitutively active STAT3 in cervical precancer and cancer lesions, whereas it was absent in normal controls. Similarly, a high level of constitutively active STAT3 expression was observed in HPV-positive cervical cancer cell lines when compared to that of HPV-negative cells. Expression and activity of STAT3 were found to change as a function of severity of cervical lesions from precancer to cancer. Expression of active pSTAT3 was specifically high in cervical precancer and cancer lesions found positive for HPV16. Interestingly, site-specific accumulation of STAT3 was observed in basal and suprabasal layers of HPV16-positive early precancer lesions which is indicative of possible involvement of STAT3 in establishment of HPV infection. In HPV16-positive cases, STAT3 expression and activity were distinctively higher in poorly-differentiated lesions with advanced histopathological grades.

**Conclusion:**

We demonstrate that in the presence of HPV16, STAT3 is aberrantly-expressed and constitutively-activated in cervical cancer which increases as the lesion progresses thus indicating its potential role in progression of HPV16-mediated cervical carcinogenesis.

## Background

As the commonest cause of cancer-related female mortality in developing countries and second most frequent women malignancy worldwide, cervical cancer is the major reproductive health problem of women globally [1]. Several molecular and clinico-epidemiological studies demonstrate persistent infection of high-risk human papillomaviruses (HR-HPV) as causative agents for the development of cervical precancer and cancer lesions [2]. Though 15 different HR-HPV types are known to infect human genital tract and are associated with malignant transformation, prevalence of HPV type 16 infection is exclusively very high and constitute about 50% of total HPV prevalence globally [3]. Interestingly, HPV16 is the most common HR-HPV type associated with cervical malignancies and is found in more than 90% of the cervical cancer cases in India [4,5]. With annual incidence of about 132,000 and mortality of 74,000 [6], India shares one fourth of global cervical cancer burden. Despite availability of two HPV vaccines directed against HPV16 and HPV18 for control of cervical cancer [7] that have been licensed for clinical application in several countries including India, it is difficult to control HPV infection through vaccination. These vaccines are not just expensive but are only prophylactic in nature and do not possess any therapeutic efficacy. To aggravate the scenario, until date there is no standard therapeutic modality available that can cure these viral infections [7]. Therefore, for effective therapeutic intervention of HPV and to prevent cervical cancer development at an early stage, it is essential to improve understanding of molecular mechanisms involved in HPV-mediated cervical carcinogenesis.

Although HPV infection is essential, it is not sufficient for ultimate tumorigenic transformation and requires certain crucial host cell factors to regulate its viral gene transcription. Expression of viral transforming genes, E6 and E7, of HR-HPVs is primarily regulated by *cis*-element rich enhancer region termed as Long Control Region (LCR) located upstream to its single early promoter p97 [8]. Apart from viral transcriptional regulator, E2, the expression of viral genes/oncogenes is controlled by host's sequence-specific transcription factors such as SP-1, AP-1 and NF-κB, that specifically bind to the LCR [9]. These transcription factors are normally modulated at the level of expression and/or their activation. Host transcription factors in association with viral factors are likely to dictate viral latency, vegetative replication or oncogenic transcription during HPV infection. Some of these transcription factors such as AP-1 and NF-κB, are found to be up-regulated and transcriptionally active during cervical carcinogenesis [5,10].

Signal Transducer and Activator of Transcription (STAT) family, an important class of broad spectrum inducible transcription factors with seven known members plays an indispensable role in normal cellular events like differentiation, proliferation, cell survival, apoptosis, and angiogenesis following cytokine, growth factor and hormone signaling [11]. Aberrant activation of STAT3, a member of STAT family, has been strongly associated with carcinogenesis and shown to promote cell cycle progression, cell proliferation and oncogenic transformation [12]. STAT3 is activated primarily through phosphorylation at Tyr705 residue [13]. However, other post-transcriptional modifications like phosphorylation at Ser727 or acetylation at Lys685 are also known to independently or simultaneously affect STAT3 activity [14,15]. Tyrosine phosphorylation is responsible for STAT3 homo- and/or hetero-dimerization and their translocation to the nucleus, where it binds to specific consensus DNA sequences within promoters of its downstream target genes known to regulate apoptosis, proliferation, metastasis, invasion and other important events during carcinogenesis [16]. In addition to initial activation by tyrosine phosphorylation, phosphorylation of STAT3 on serine residue 727 maximally activates its transcriptional activity [14]; whereas, STAT3 acetylation is responsible for stabilization of this multi-protein-DNA complex [15,17].

Aberrant expression/activation of STAT3 has been observed in a wide number of human cancer cell lines and primary tumors including blood cancers and solid tumors and has been shown to be associated with the poor prognosis in various types of malignancies [18]. Though some studies demonstrate presence of STAT3 in a subset of cervical lesions [19-22], not much is known about the expression and activation of STAT3 during cervical carcinogenesis in general, and its relation to HPV infection, in particular. Recently, a potential STAT3 binding site has been mapped on to 5' region of HPV16 LCR that controls expression of viral oncogenes [23], thus suggesting a plausible productive interaction between HPV infection and STAT3 signaling.

The cervical cancer provides a unique window of opportunity for studying the expression of important markers of disease progression as the tumorigenic transformation of cervical epithelial cells takes 10-15 years to occur. Cervical carcinogenesis progresses through histopathologically well-characterized precursor lesions. Keeping in view the above advantages, present study was designed to evaluate the expression pattern of STAT3, its phosphorylation and cellular distribution, and DNA-binding activity in different grades of cervical precancer and cancer lesions in relation to HPV16 infection to understand the involvement of STAT3 in HPV16-induced cervical carcinogenesis.

## Materials and methods

### Cell lines and Clinical Specimens

Established cervical cancer cell lines C33a (HPV^-^), SiHa and CaSki (HPV16**^+^**) and HeLa (HPV18**^+^**) cells free of intra/inter species cross-contamination were procured from ATCC and were maintained in prescribed culture conditions. A total of 120 fresh cervical tissue biopsies were collected comprising 70 malignant, 30 premalignant and 20 normal (control) cervical tissues prior to any chemo/radio therapy from the Cancer Clinic, Gynae Out Patient Department of Lok Nayak Hospital, New Delhi, India. Written informed consent was obtained from all the subjects included in the study and was carried out in accordance with the principles of the Helsinki Declaration and clinico-epidemiological details were taken from their clinical records. The study was approved by the Institutional Ethics Committee. The clinico-epidemiological characteristics are presented in Table 1. A portion of each biopsy collected in cold 1× phosphate buffer saline (PBS) was immediately processed for molecular biological works and the other half was sent for histopathological diagnosis in formalin solution. All reagents used in the study were of analytical or molecular biology grade and procured from Sigma Aldrich (USA) unless specified.

**Table 1:** Clinico-pathological characteristics and HPV status of cervical biopsies from normal control tissues and from cervical precancer and cancer lesions

Tissue type	Characteristics	Number of samples	Total HPV+ (HPV L1)	HPV 16+ (% positivity)	Mean age (Years; ± SD)
**Normal controls**		**20**	**0**	**0**	
	Normal cervical tissues without inflammation	15	0	0	25 ± 4.6
	Normal cervical tissues with inflammation	5	0	0	31 ± 3.5
**Precancer**		**30**	**17**	**16 (53%)**	**29 ± 3.5**
	Low grade squamous intraepithelial lesions	15	7	7 (47%)	
	High grade squamous intraepithelial lesions	15	10	9 (60%)	
**Cancer Tissues**		**70**	**65**	**58 (83%)**	**50 ± 12.6**
***Histopathological grading***	Well Differentiated SCC	35	32	30 (86%)	
	Moderately Differentiated SCC	25	23	20 (80%)	
	Poorly Differentiated SCC	10	10	8 (80%)	
					
***Clinical stages***	Stage I	08	06	05 (63%)	
	Stage II	18	15	13 (72%)	
	Stage III	32	32	30 (94%)	
	Stage IV	12	12	10 (83%)	

HPV - Human papillomavirus, SCC - Squamous cell carcinoma

### Isolation of DNA and diagnosis of HPV infection

High molecular weight genomic DNA was isolated from normal, precancerous and cancerous cervical biopsies by the standard proteinase K digestion and phenol-chloroform extraction procedure, and PCR amplification was performed following the procedure described earlier [4]. The initial HPV diagnosis was performed by using a pair of consensus degenerate primers (MY09 and MY11) derived from the highly conserved L1 open reading frame of HPV genome (MY09:5'-GCMCAGGGWCATAAYAATGG-3', MY11:5'-CGTCCMARRGGAWACTGATC-3' where M = A/C; W = A/T; Y = C/T; R = A/G). HPV16 and HPV18 typing was done by type-specific primers [HPV16-(F) 5'-AAGGCCAACTAATAGTCAC-3', (R) 5'-CTGCTTTTATACTAACCGG-3'; HPV18-(F) 5'-ACCTTAATGAAAAACCACGA-3', (R) 5'-CGTCGTTTAGAGTCGTTCCTG-3']. PCR was performed in a 25 μL reaction mixture containing 50-100 ng DNA, 10 mM Tris-HCl (pH 8.4), 50 mM KCl, 1.5 mM MgCl_2_, 125 mM of each dNTPs (dATP, dGTP, dCTP, dTTP), 5 pmol of oligonucleotide primers and 0.5 U Taq DNA polymerase. β-globin gene was used as internal control [(F) 5'-CAACTTCATCCACGTTCACC-3', (R) 5'-GAAGAGCCAAGGACAGGTAC-3')]. The temperature profile used for amplification constituted an initial denaturation at 95°C for 4 min followed by 30 cycles of denaturation at 95°C for 30 sec, annealing at 55°C for 30 sec and extension at 72°C for 1 min, which was extended for 5 min at the final cycle. Custom-synthesized, HPLC-purified primers were procured from M/s Microsynth (Germany).

### Isolation of total, cytoplasmic and nuclear proteins from cervical tissues and cell lines

Total proteins from biopsies were prepared by the method described previously [24]. Briefly, the method involved fine mincing of either fresh or frozen biopsies stored at -80°C, in cold 1× PBS with surgical blade in petridish on ice. The minced tissue material was later centrifuged at 4,000 rpm at 4°C to wash off 1× PBS solution. For preparation of total proteins, the pellet from minced tissue or different cell lines (2×10^6 ^cells) was re-suspended in lysis buffer (20 mM Tris (pH 7.4), 250 mM NaCl, 2 mM EDTA (pH 8.0), 0.1% Triton X-100, 0.01 mg/ml aprotinin, 0.005 mg/ml leupeptin, 0.4 mM PMSF, and 4 mM Na_3_VO_4_) as described previously [25]. Lysates were spun at 14,000 rpm for 10 min to remove insoluble material. Alternatively, for preparation of cytoplasmic proteins, the cell/minced tissue pellet was resuspended in ice-cold bufferA [20 mM HEPES pH = 7.6, 20% (v/v) Glycerol, 10 mM NaCl, 1.5 mM MgCl_2_, 0.2 mM EDTA, 1 mM DTT, 1 mM PMSF, 2 mg/ml Leupeptin and 10 mg/ml Aprotinin] and incubated on ice for 10 min with frequent vortexing. Lysate was centrifuged at 4000 rpm for 10 min at 4°C to obtain supernatant as cytoplasmic proteins. The remaining pellet containing isolated nuclei was re-suspended in bufferB [20 mM HEPES pH 7.6, 25%(v/v) Glycerol, 500 mM NaCl, 1.5 mM MgCl_2_, 0.2 mM EDTA, 1 mM DTT, 1 mM PMSF, 2 mg/ml Leupeptin and 10 mg/ml Aprotinin] and centrifuged after incubation for 1 hr with repeated vortexing on ice at 14,000 rpm for 25 min at 4°C to obtain supernatant designated as nuclear proteins. The concentration of proteins was determined by spectrophotometric method and the proteins were stored in aliquots at -80°C till further use.

### Electrophoretic Mobility Shift Assay (EMSA)

For analysis of STAT3 DNA binding activity in tissues or cell lines, nuclear proteins were checked by EMSA as described earlier [26]. Briefly, 10 μg of nuclear proteins from each sample were incubated with **^32^**P-radiolabeled oligonulceotide probe containing a hSIE derived from the c-fos gene promoter (sense strand, 5'-AGCTTCATTTCCCGTAAATCCCTA-3') that binds activated STAT3 proteins [27]. Protein DNA complexes were resolved by non-denaturating PAGE (6%). The gel was dried and detected by Phosphoimager (FLA-5100, Fujifilm, Japan). Quantification of STAT3 activation levels was performed using Alpha Ease FC version 4.1.0 (Alpha Innotech Corporation, IL). Human anti-pSTAT3 specific to Phospho Tyr705 or specific to Phospho Ser727 (BD Biosciences, USA) were used to identify STAT3 specific DNA-proteins complexes in a super-shift assay. For supershift analysis, 2 μg of each pSTAT3 antibody was incubated with nuclear proteins for 20 min at room temperature before the addition of radiolabeled probe and electrophoresis. Binding specificity of STAT3 probe was checked by using nuclear proteins of SiHa cells with unlabelled 100× molar excess of cold specific competitor (STAT3 probe) or mutant STAT3 oligo (data not shown) and non-specific competitor (Oct-1).

### Immunoblotting

Total cellular proteins (50 μg/lane) were separated in 8-12% polyacrylamide gel and electrotransferred on PVDF membranes (Millipore Corp, Bedford, MA, USA). The membrane was blocked in PBS containing 5% non-fat skimmed milk and probed with specific antibody by incubating the membrane overnight in pre-standardized dilution of primary antibody in blocking solution at 4°C. These blots were washed, incubated with HRP-anti-mouse IgG secondary antibodies and visualized by Luminol detection kit (Santa Cruz Biotech, USA) and by exposing the blot to KODAK X-Omat films (Kodak India, India). The western blot membranes were re-probed for β-actin expression as an internal control. The quantitative densitometric analysis of the bands was performed using Alpha Ease FC version 4.1.0 (Alpha Innotech Corporation, IL). The expression level of proteins was quantitated on an arbitrary scale with respect to β-actin expression where Strong (+++) - > 50%; Medium (++) - 10-50%; Weak (+) - < 10% of β-actin expression; and Nil (-) - not-detectable. pSTAT3/STAT3 ratios were determined by assessing the densitometric analysis of bands visualized in immunoblot and normalized to β-actin expression.

### Immunohistochemistry

The immunohistochemical staining was performed as described previously [24]. Briefly, 5 μm section of freshly fixed and paraffinized tissue sections were deparaffinized, rehydrated and subjected to heat-induced epitope retrieval in the 10 mM citrate buffer (pH 6.0). Non-specific binding sites were blocked using 1.5% blocking serum and incubated overnight in pre-standardized dilution of primary antibody. Immunoreactivity was visualized according to manufacturer protocol (ABC staining kit, Santa Cruz Biotech).

### Histopathological and immunohistochemical evaluation

The Bethesda system of classification was used in the present study for the histopathological grading of cervical precancer and cancer cases [28]. Two independent pathologists performed the histopathological evaluation of hematoxylin and eosine-stained tissue sections as per routine procedure. Scoring of IHC staining was performed by three independent investigators (SS, SH and ACB). In case of sections from normal tissues and LSILs, basal and suprabasal epithelial cells were analyzed for STAT3/pSTAT3 immunostaining; whereas for HSILs, basal, suprabasal, and intermediate layers of epithelial cells were analyzed. In cancer cases, all the cells were analyzed for STAT3/pSTAT3 immunostaining. Overall inter-observer difference varied between 5-10%. Discrepant scores were resolved by joint evaluation and consensus. Every IHC-stained tissue was scored as reported earlier [24,29] on an arbitrary scale according to the number of positively stained cells and overall staining intensity of the section and assigned a value ranging from Nil (-) - no staining; Weak (+) - 1-10% cells showing focal positivity; Moderate (++) - 11-50% cells showing focal positivity; and, Strong(+++) - more than 50% cells showing diffused positivity.

### Analysis of STAT3 mRNA expression by reverse transcription-PCR

Total RNA was isolated from cervical cancer cell lines and from a subset of cervical normal (n = 5), precancer (n = 8) and cancer tissues (n = 10) by TRI reagent(r) as per manufacturer's instruction (Sigma-Aldrich Inc, USA). The quality and integrity of RNA was checked spectrophotometrically and on 1.0% agarose gel. For reverse transcription-PCR (RT-PCR) analysis, 3 μg of total RNA was subjected to reverse transcription using Omniscript RT-PCR kit (Qiagen, USA) to prepare the cDNA. For the amplification of STAT3 mRNA, the primer pairs 5'-TTGCCAGTTGTGGTGATC-3'and 5'-GAACCCAGAAGGAGAAGC-3' were used with the following conditions: 94°C for 5 min, then 30 cycles of 94°C for 30 sec, 55°C for 30 sec, and 72°C for 1 min, followed by 72°C for 5 min that resulted in amplicon of 318 bp size [30]. Amplification of GAPDH (forward 5'-TGGATATTGTTGCCATCA ATGACC-3'; reverse 5'-GATGGCATGGACTGTGGTCATG-3') transcripts from the respective cDNAs was used as an internal control (520 bp). The PCR products were analyzed on 3% agarose gel.

### Statistical analysis

The data analysis was performed using the statistical software Sigma Stat Graph Pad Instat (version 4.0). Fisher's Exact Test (for smaller numbers on subgroup analysis), t-test or chi square were used to compare the expression of proteins among different histopathological grades of tissue biopsies. *p *values of < 0.05 were considered statistically significant.

## Results

Established cervical cancer cell lines (C33a, SiHa, CaSki, HeLa) and 100 prospectively collected biopsies from precancer and cancer lesions and 20 control specimens from histopathologically-confirmed, normal hysterectomized cervix were examined for presence of HPV infection as well as expression and activation of STAT3. Clinico-pathological characteristics, demographical details of the cases and controls and status of HPV infection in corresponding tissues is presented in Table 1.

### Detection of HPV DNA sequences in cervical precancer and cancer tissues

To determine the status of HPV infection and distribution of HPV16/18 types in study samples, HPV L1 and type-specific PCRs were performed as described in Methods. PCR-based detection revealed HPV infection in 17 out of 30 cases (57%) of precancer lesions and 65 out of 70 cases (92%) of cancer lesions (Table 1). All normal control tissues were confirmed HPV negative. Out of 82 tissues positive for HPV L1 sequences, 13 out of 17 precancer (76%) and 58 out of 65 cancer (89%) tissues were found to contain HPV16 infection, whereas, in 1 (6%) precancer and 9 (13%) cancer cases HPV18 or other high-risk HPV infections were detected. Distribution of HPV16 positive cases was similar across different histopathological grades and clinical stages.

### STAT3 expression and phosphorylation in cervical cancer cell lines and tumor biopsies

**Figure 1:**
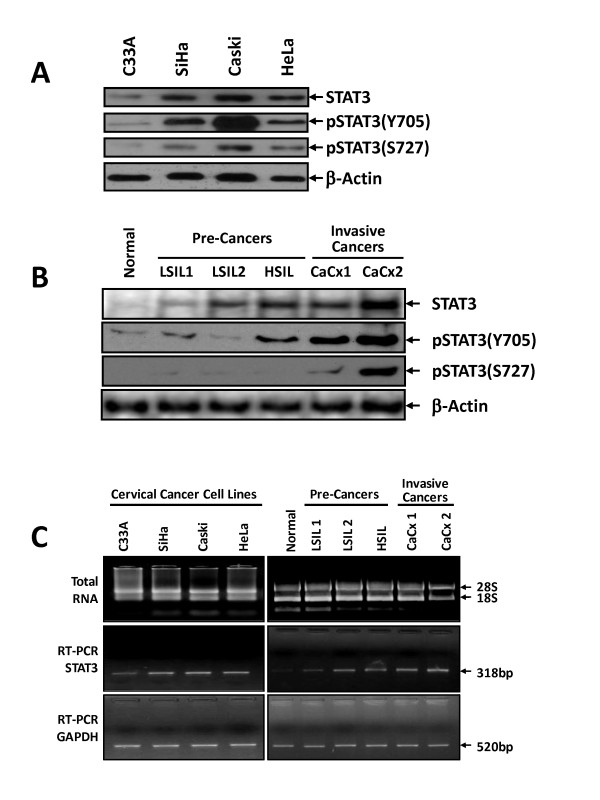
**Cervical cancer cells over-express STAT3 with phosphorylation on both tyrosine and serine residues**. (A) Cervical cancer cell lines C33a (HPV^-^), SiHa & CaSki (HPV16**^+^**) and HeLa (HPV18**^+^**) (2 × 10^6 ^cells) were lysed, and 50 μg of total cellular proteins were resolved on 7.5% SDS-PAGE, electrotransferred to a PVDF membrane, and probed for STAT3, pSTAT3(Y705), pSTAT3(S727) and β-actin expression by respective antibodies. (B) STAT3 expression and phosphorylation increases as a function of severity of cervical lesions. Representative immunoblot of STAT3, pSTAT3(Y705), pSTAT3(S727) and β-actin indicating expression of respective protein in total cellular proteins (50 μg) isolated from normal (N), low grade squamous intra-epithelial lesion (LSIL1 & 2), high grade SIL (HSIL) and cervical cancer biopsy tissues (CaCx1, CaCx2) as described in Methods. (C) STAT3 RT-PCR analysis of cDNA derived from cervical cancer cell lines and cervical tissues. Representative ethidium bromide-stained agarose gel (3%) showing specific amplification STAT3 transcripts (318 bp) in the cDNA derived from total RNA of indicated samples (middle panel). Quality and quantity of RNA used for cDNA preparation was examined and confirmed by 1% agarose gel electrophoresis (upper panel). GAPDH RT-PCR (amplicon size 520 bp) was used as internal control (lower panel).

To determine STAT3 expression and its phosphorylation at Y705 and S727 residues in cervical cancer, HPV positive [SiHa & CaSki (HPV16**^+^**) and HeLa (HPV18**^+^**)] and HPV^- ^cervical cancer cell lines (C33a) were tested for expression of STAT3 and pSTAT3 (Y705 & S727) by immunoblotting. As shown in Figure 1A, immunoblot analysis of cellular proteins demonstrated constitutive STAT3 expression in all cervical cancer cell lines although the degree of expression was variable. In contrast to HPV^- ^C33a cells, all HPV positive cells had higher expression of STAT3. These cellular proteins were simultaneously tested for expression of phosphorylated STAT3 at Y705 & S727 by monoclonal antibodies that could distinguish between the two phosphorylated forms of STAT3 from each other. Interestingly, all four cell lines irrespective of HPV infection expressed STAT3 phosphorylated at Y705 & S727 residues, though HPV16**^+ ^**cells expressed a comparatively higher level of phosphorylated forms in comparison to HPV^- ^(C33a) or HPV18**^+ ^**(HeLa) cells.

Further, we examined the expression and phosphorylation of STAT3 in cervical precancer and cancer tissues and compared with normal controls. As shown in Figure 1B and (Additional file 1: **Table S1**), only trace amounts of STAT3 were expressed in normal cervical tissues whereas both low grade as well as high grade precancer lesions (LSIL and HSIL) expressed either moderate or high levels of STAT3 respectively. STAT3 was consistently over-expressed in cancerous tissues (Additional file 1: **Table S1**). Immunoblotting for pSTAT3(Y705) and pSTAT3(S727) revealed a concordant increase in pSTAT3 levels in precancer and cancer lesions indicating that STAT3 expressed in these lesions was phosphorylated both at Y705 and S727 residues. The expression of STAT3 and its phosphorylated forms was found to increase as a function of severity of the cervical lesions from precancer to cancer stages.

To assess whether STAT3 is also elevated at mRNA level, the total RNA isolated from cervical cancer cell lines or from a subset of cervical tissues comprising normal, precancer (LSIL and HSIL) and cancer lesions were subjected to cDNA synthesis and STAT3-specific RT-PCR. As shown in Figure 1C, both HPV positive cervical cancer cell lines as well as HSIL and cancer tissues expressed high levels of STAT3 transcripts. On the other hand, level of STAT3 transcript was moderate in HPV negative cell line C33a and LSILs while it was not detectable in normal tissues.

### Increased expression and nuclear localization of STAT3 and phosphorylated STAT3 in cervical precancer and cancer lesions

**Figure 2:**
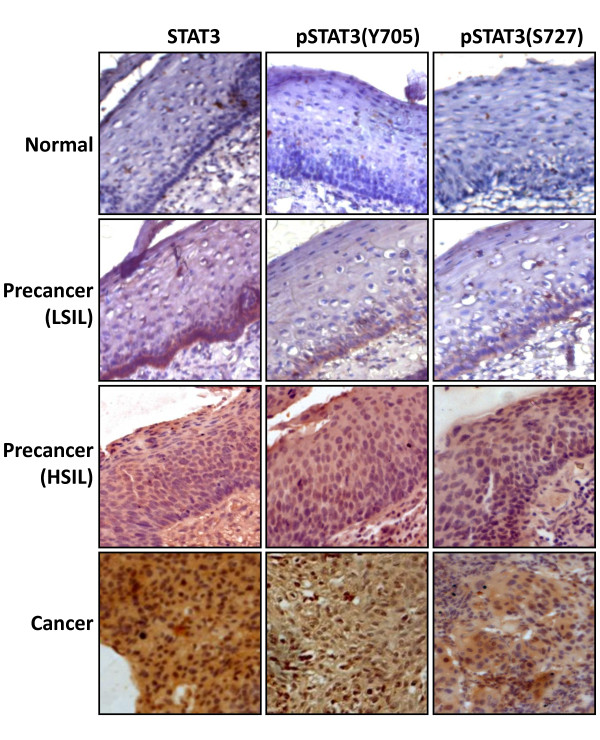
**Increased expression and nuclear localization of STAT3 and pSTAT3 in cervical precancer and cancer lesions**. Representative photomicrographs of immunohistochemical analysis of STAT3, pSTAT3(Y705) and pSTAT3(S727) in normal, precancer (LSIL & HSIL) and cancer lesions. Freshly fixed, paraffin-embedded sections (5 μm) of cervical tissues from normal, precancerous and cancerous lesion of the cervix were processed for IHC and probed for STAT3, pSTAT3(Y705) and pSTAT3(S727) with respective antibodies and detected by HRP-DAB method as described in Methods. Brown precipitate indicates immuno-positive cells, blue stain represent nuclei and co-localization of brown and blue stain represents nuclear localization of STAT3 and pSTAT3. (Original magnification: 200×)

Though immunoblotting provides an average data, a potential pitfall of the analysis using tissue samples is contribution of "contaminating" cells (e.g. stromal and inflammatory cells) to the level of STAT3 or pSTAT3 expression. Therefore, to evaluate micro-heterogeneity in the expression and cellular distribution of STAT3 in precancer and cancer tissues *in situ*, immunohistochemical protocol was optimized for analysis of STAT3, pSTAT3(Y705) and pSTAT3(S727) in freshly-collected, paraffinized tissue sections. As indicated in Figure 2 and (Additional file 2: **Table S2**), immunohistochemical analysis demonstrated complete absence of phosphorylated (Y705 or S727) or non-phosphorylated STAT3 in normal tissues (Figure 2, upper panel), however, a few control tissues with inflammatory cytology demonstrated low or moderate immunopositivity for STAT3 and pSTAT3 (data not shown). Majority of the low grade precancer lesions (LSIL) had low or undetectable STAT3 or pSTAT3 expression, though in some LSIL, STAT3 and pSTAT3 showed focal positivity in both basal and suprabasal layers that was found to be equally distributed among nuclei and cytoplasm (Figure 2, middle panels). In contrast, high grade precancer lesions (HSIL) had variable level of STAT3 and pSTAT3 expression that frequently localized to the nuclei. Of 50 cancer biopsies examined for immunohistochemical analysis of STAT3, 42 were found positive for STAT3 and out of these, 71% had either moderate or high STAT3 expression and had variable degree of nuclear positivity [(Figure 2, lower panels) and (Additional file 2: **Table S2**)]. Moreover, low levels of STAT3, pSTAT3(Y705) and pSTAT3(S727) were also detectable in the cytoplasm. Interestingly, STAT3 and pSTAT3(Y705) expression as well as nuclear localization were concordant in majority of cancer tissues, however, degree of pSTAT3(S727) expression and its nuclear localization, in general, was lower than that of STAT3 that could be attributed to variability in affinity of different antibodies.

### Constitutive activation of STAT3 in cervical cancer cell lines and tumor biopsies

**Figure 3:**
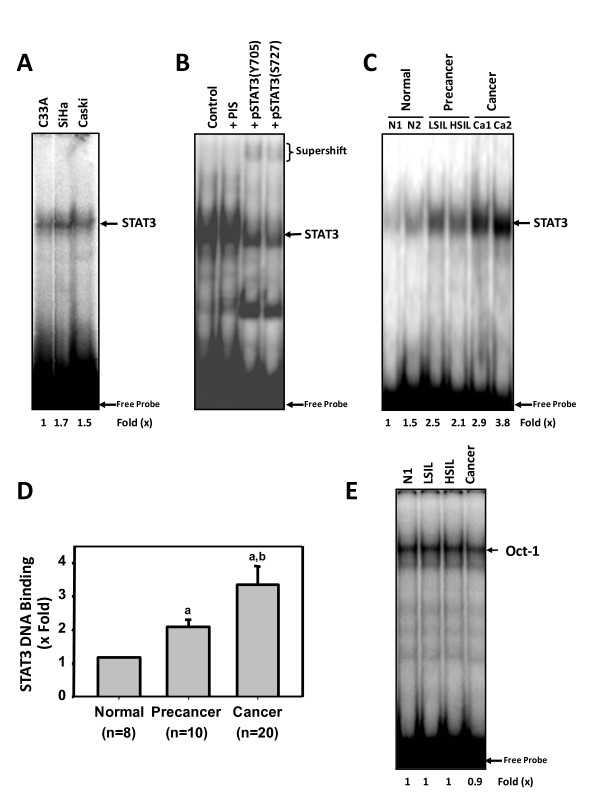
**STAT3 is constitutively active during cervical carcinogenesis**. (A) Cervical cancer cell lines C33a, SiHa and CaSki express constitutively active STAT3 DNA-binding activity. Ten μg of nuclear proteins of respective cell line were incubated with ^32^P-radiolabeled STAT3 probe and resolved on 6.0% non-denatunating PAGE. (B) Gel supershift assay showing specificity of STAT3 binding. SiHa cell nuclear proteins (10 μg) were incubated for 30 min with pSTAT3(Y705) or pSTAT3(S727) antibodies (2 μg each) or pre-immune serum (PIS) and assayed for STAT3 binding activity by EMSA. STAT3-specific complex and super-shifted bands after antibody addition is indicated. (C) Constitutive STAT3 activation in cervical precancer and cancer lesions. Nuclear proteins (10 μg) prepared from normal (N1 & N2*), precancer (LSIL and HSIL) and cancer lesions (Ca1 & 2) were tested for presence of STAT3 DNA binding activity by EMSA. The extent of STAT3 activity increased as a function of severity of the disease. Cancerous lesions showed high STAT3 binding activity. N2* showed inflammatory cytology. (D) Nuclear proteins were simultaneously checked for equal loading using EMSA for constitutively active transcription factor Oct-1 that showed uniform binding in different tissues types. (E) Mean fold change in STAT3 DNA binding activity in cervical precancer and cancer lesions with respect to normal controls. Error bars indicate standard deviation. ^a^*p *value < 0.001 versus normal control tissues; ^b^*p *value < 0.001 versus precancer tumor tissues as determined by t-test.

To evaluate DNA binding activity of overexpressed, nuclear STAT3/pSTAT3 and to confirm its constitutive activation in cervical cancer, we performed EMSA analysis of nuclear proteins derived from human cervical cancer cell lines, C33a, SiHa and CaSki and a subset of freshly collected biopsies from normal, precancer and cancer lesions. Figure 3A, shows presence of active STAT3 complexes in both HPV negative and HPV16 positive cervical cancer cell lines. Compared to HPV^- ^C33a cells, HPV16^+ ^SiHa and CaSki cells showed marginally higher DNA-binding activity. Binding of hSIE probe to STAT3 was found to be specific as antibody to pSTAT3 could specifically bind and supershift retarded DNA-protein complex, confirming that the activity corresponds to STAT3 (Figure 3B). On the other hand, normal cervical tissues expressed low or undetectable STAT3 DNA binding activity except in a few cases that had inflammatory cervix (Figure 3C, N1 and N2 respectively). In comparison, precancer lesions (both LSIL and HSIL) showed moderate STAT3 DNA binding, whereas, cancer biopsies revealed a prominent STAT3 activity (Figure 3D). When Oct-1 was used as a probe, which served as a control, there was no difference in DNA binding activity between normal, LSIL/HSIL or cancer (Figure 3E), thus indicating that increased expression and activation of STAT3 is specific to the process of cervical carcinogenesis.

### Association of HPV16 infection with STAT3/pSTAT3 expression in cervical precancer and cancer lesions

**Figure 4:**
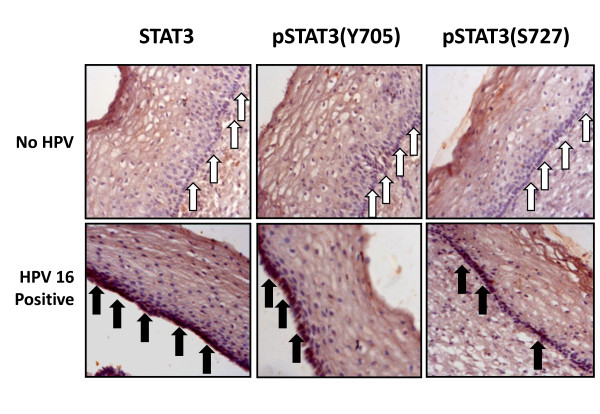
**Localized overexpression of STAT3 and pSTAT3 in basal layer of early precancer lesions positive for HPV16**. Immunohistochemical analysis of STAT3, pSTAT3(Y705) and pSTAT3(S727) expression in HPV negative and HPV16 positive LSILs. White arrows indicate absence of STAT3 expression in basal layer of HPV negative lesions. STAT3 and pSTAT3 showed focal positivity and were localized in the nuclei in HPV16 positive LSIL tissues (marked by black arrows).

To analyze the effect of HPV16 infection on STAT3 expression and activity in different stages of cervical carcinogenesis, the STAT3/pSTAT3 immunoblotting data of precancer and cancer lesions were reanalyzed with respect to the status of HPV infection in the respective lesion. As shown in Table 2, HPV16^+ ^cervical precancer and cancer tissues expressed a higher level of STAT3 and pSTAT3(Y705) as compared to that in the HPV^- ^precancer and cancer lesions. One precancer (HSIL) and 7 carcinoma samples that were HPV L1^+ ^but HPV16^- ^were excluded from the analysis to avoid HPV type as a confounding variable. Nonetheless, these tissues also showed similar over-expression of active pSTAT3/STAT3. Majority of HPV^- ^precancer, cancer and normal tissues lacked expression of STAT3 and pSTAT3 while only a small number of HPV16^+ ^precancers (n = 6/16; 37%) and cancers (n = 12/58; 21%) had no or low STAT3 expression. Interestingly, immunohistochemical analysis of precancer lesions particularly of LSILs showed a focal positivity of STAT3 and when these cases were analyzed with respect to their HPV16 status they showed a low background staining with no nuclear positivity for STAT3 as well as pSTAT3 in HPV negative LSIL sections (Figure 4). In contrast, HPV16 positive LSIL sections revealed a strong focal positivity and nuclear localization of both STAT3 and pSTAT3 (Y705 & S727) in basal and suprabasal cell layers of cervical epithelium.

**Table 2:** Expression of STAT3 and phosphorylated STAT3 [pSTAT3(Y705) and pSTAT3(S727)] in HPV^- ^and HPV16^+ ^precancer and cancer lesions of the uterine cervix as observed by immunoblotting^1^

Target Protein	Normal (n = 20)		Precancer (n = 29)				Cancer (n = 63)				*p *value
	**HPV**^**- **^**(n = 20); (%)**		**HPV**^**- **^**(n = 13); (%)**		**HPV16**^**+ **^**(n = 16); (%)**		**HPV**^**- **^**(n = 5); (%)**		**HPV16**^**+ **^**(n = 58); (%)**		
	
Expression Level (→)	N(-)/W(+)	M(++)/S(+++)	N(-)/W(+)	M(++)/S(+++)	N(-)/W(+)	M(++)/S(+++)	N(-)/W(+)	M(++)/S(+++)	N(-)/W(+)	M(++)/S(+++)	
**STAT3**	18 (90%)	2 (10%)	12 (92%)	1 (8%)	6 (37%)	10 (63%)	4 (80%)	1 (20%)	12 (21%)	46 (79%)	**0.005^a^, 0.01^b^, **0.49^c^, 0.19^d^
**pSTAT3 (Y705)**	19 (95%)	1 (5%)	12 (92%)	1 (8%)	8 (50%)	8 (50%)	4 (80%)	1 (20%)	21 (36%)	37 (64%)	**0.01^a ^**0.07^b^ 0.49^c^ 0.47^d^
**pSTAT3** **(S727)**	19 (95%)	1 (5%)	12 (92%)	1 (8%)	8 (50%)	8 (50%)	4 (80%)	1 (20%)	28 (48%)	30 (52%)	0.11^a^ 0.37^b^ 0.49^c^ 0.87^d^

^1^Arbitrary level of expression in immunoblotting: Strong (S) = (+++); Medium (M) = (++); Weak (W) = (+); Nil/not detectable (N) = (-). Values indicate the distribution of specimens in each category.

*p *value, probability from Fischer's Exact Test comparing the expression of proteins; ^a ^HPV^- ^vs HPV16^+ ^precancer cases, ^b ^HPV^- ^vs HPV16^+ ^cancer cases, ^c ^HPV^- ^Precancer vs HPV^- ^in cancer cases, ^d ^HPV16^+ ^Precancer vs HPV16^+ ^cancer cases.

### Differential expression and activation of STAT3 in various histopathological grades of the HPV16 positive cancer lesions

**Figure 5:**
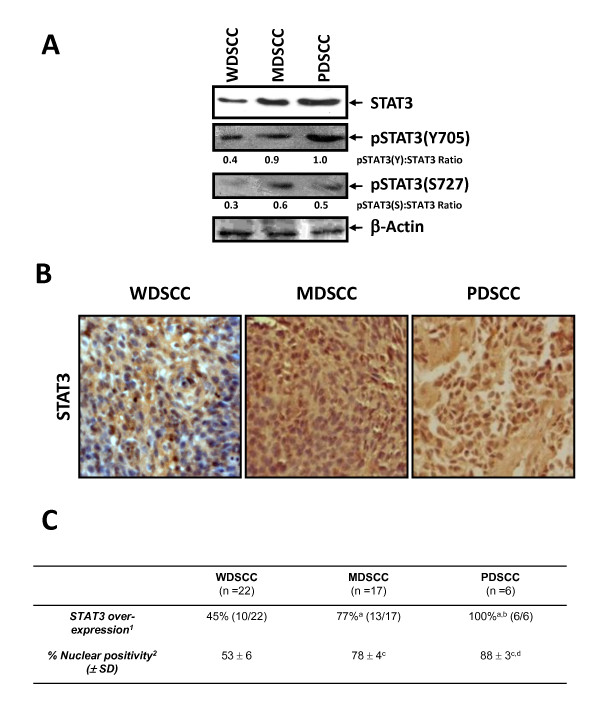
**Differential expression of STAT3 and pSTAT3 in various histopathological grades of the HPV16 positive lesions during cervical carcinogenesis**. (A) Representative immunoblots showing expression pattern of STAT3 and pSTAT3 proteins in well differentiated (WDSCC), moderately differentiated (MDSCC) and poorly differentiated (PDSCC) squamous cell carcinoma nuclear proteins with densitometric ratio of pSTAT3: STAT3 expression. (B) Representative photomicrographs showing differential expression and nuclear localization of STAT3 on paraffin embedded sections of HPV16^+ ^invasive cervical cancer tissues in various histopathological grades. C. Cumulative data of immunohistochemical evaluation of degree of STAT3 over-expression and nuclear localization which increases as a function of severity of the disease. ^1^Values indicate percentage of specimens showing either medium or strong overall STAT3 expression on an arbitrary staining intensity grading as described in Methods. ^2^Percent number of cells showing nuclear positivity in specimen expressing medium or strong STAT3 expression. *p *value < 0.001 versus values for WDSCC^a ^or MDSCC^b ^as determined by Fischer's Exact Test/Chi Square test. *p *value < 0.001 versus values for WDSCC^c ^or MDSCC^d ^as determined by t-test.

Since STAT3 expression/activation was localized in HPV16^+ ^precancer lesions. To determine its correlation we examined STAT3 expression in HPV16^+ ^cervical cancer cases with different histopathological grades. Forty five (n = 45) cancer biopsies with confirmed histopathology and HPV16 positivity were re-evaluated for STAT3 and pSTAT3 expression. As shown in Figure 5A and 5B, a comparative immunoblotting and immunohistochemical analysis revealed a lower expression of STAT3 and pSTAT3 (both Y705 and S727) in WDSCC cases in comparison to MDSCC and PDSCC that had high STAT3 and pSTAT3 expression. Elevated level of pSTAT3 in MDSCC and PDSCC were also corroborated with intense nuclear positivity of STAT3 in histologically-advanced cancer tissues and was found in as high as 78% and 88% of cancer cells in MDSCC and PDSCC respectively. In contrast, only in 53% of cells in WDSCC sections showed nuclear localization of STAT3 proteins (Figure 5C). Together, these findings indicate that constitutive activation of STAT3 is a frequent occurrence in high grade malignant cervical carcinomas and positively correlated with poorer histopathological grades.

## Discussion

In the present study, we demonstrate aberrantly expressed and constitutively active STAT3 both in cervical cancer cell lines and in cervical precancer and cancer lesions. Expression of STAT3 was elevated at transcript level and was found associated with simultaneous increase in phosphorylation at both, Tyr705 and Ser727, that are known to regulate STAT3 dimerization, nuclear transport, DNA-binding and transactivation. Dually phosphorylated STAT3 present in cervical precancer and cancer lesions was found to localize to the nuclei and possessed a functional DNA-binding activity. Our immunoblotting, IHC and DNA binding assays revealed that aberrant STAT3 activity increases as a function of severity of the disease from precancer to cancer during cervical carcinogenesis and was found associated with HPV16 positive lesions. Moreover, STAT3 expression and activation correlated well with HPV16 positivity in cervical precancer and cancer lesions which indicates its possible involvement in establishment of HPV infection and persistence. In addition, when correlated with different histopathological grades in HPV16 positive cancer lesions, cases with more advanced histopathological grades had significantly higher expression of active STAT3.

In precancer lesions, we observed STAT3 immunopositivity ranging from 10 to 25% by IHC and about 33-40% by immunoblotting. STAT3 positivity, irrespective of the technique, was consistently higher than pSTAT3(Y705) and pSTAT3(S727) indicating a smaller proportion of STAT3 pool could be in unphosphorylated state. Similarly, pSTAT3(S727) was under-represented indicating potentially lesser phosphorylation at serine residue. However, validity of such claim(s) cannot be ascertained, as stoichiometry of antibody-binding is likely to be variable due to difference in affinity of various STAT3 antibodies. Alternately, it is possible that a discrepantly larger proportion of tumors showing high level of STAT3 but with lower pSTAT3 levels may have a major pool of STAT3 that could be in non-phosphorylated state and might be activated by alternate mechanisms like acetylation at Lys685 which reportedly activates STAT3's sequence-specific DNA-binding and subsequent activation independent of tyrosine phosphorylation [15,17]. Our observations indicating a potential role of STAT3 in cervical carcinogenesis have been supported by similar results from others demonstrating presence of pSTAT3 in nuclei of cervical neoplastic cells, though with variable levels of expression ranging from 24% to 56% [19-22] (Table 3). These studies were primarily conducted using pSTAT3 IHCs on retrospectively collected specimens. About 40% STAT3 nuclear positivity has been demonstrated in cervical precancer lesions [21,22], which predominantly were of HSILs category (CIN2 and CIN3). Our results show a higher STAT3 positivity in HSIL compared to LSIL (CIN1) type of precancer lesions, but the difference was not statistically significant (data not shown). In contrast to moderate immunopositivity in precancer lesions, more than 70% cancer biopsies examined expressed moderate to high levels of STAT3 which was supported by both pSTAT3 IHC as well as immunoblotting. Earlier reports showed presence of pSTAT3 in cervical cancer tissues albeit to a much lesser level (24%) [19] that could be due to the use of preserved tissue blocks or tissue arrays. Similarly, in a recent report, with lower stringency of analysis by considering 10% cells expressing nuclear pSTAT3 as cut off for positives, a maximum of 57% STAT3 positivity was observed in cervical cancer tissues [20]. A higher STAT3 expression and activation found in our study could be either due to the variations in histologic subtype, definition of positive expression or the improved quality of sample as they were processed immediately. STAT3 being a labile protein and since the specimen for earlier studies were archived samples, it is quite likely that due to prolonged storage STAT3 might have undergone degradation or dephosphorylation which may in part be responsible for the low levels of pSTAT3 expression. Moreover, the studies reported earlier had used only IHCs for pSTAT3 and did not examine total STAT3 expression simultaneously. It is important to note that phosphorylation level of STAT3 is critically influenced by the processing time following tissue resection. Prolonged delay in the fixing or freezing of tissue specimens has been shown to reduce phosphorylation level of STAT3 due to the activity of tyrosine phosphatases [31]. Moreover, all earlier studies did not correlate STAT3 expression in cervical lesion with the status of HPV infection, the principle agent that cause cervical cancer. Increased STAT3 expression/activation in present study may also be associated with geographical variations in the spectrum of incident HPV infection. As reported earlier [4], HPV16 is the most prevalent HR-HPV type in cervical cancer of Indian women and in present study also we observed a high frequency of HPV16 sequences in about 83% of cancers and 53% in precancer tissues. This is much higher prevalence of HPV16 in cervical cancer than that from other Asian countries which ranges from 45%-52% [32,33].

**Table 3:** Comparative analysis of studies examining expression of STAT3 and phosphorylated STAT3 in cervical precancer and cancerous lesions

Study (Year)	Tissues		Type of study	Immunohistochemistry (% positivity)			Immunoblotting (% positivity)			STAT3 DNA Binding	Correlation with HPV positivity
					
	Precancer (n)	Cancer (n)		STAT3	pSTAT3 (Y705)	pSTAT3 (S727)	STAT3	pSTAT3 (Y705)	pSTAT3 (S727)		
Yang et al. (2005)	56	-	Retrospective	ND	ND	42% (24/56)^a^	ND	ND	ND	ND	ND
Yang et al. (2006)	56	-	Retrospective	39% (22/56)^a^	ND	ND	ND	ND	ND	ND	ND
Chen et al. (2007)	-	104	Retrospective	ND	24% (25/104)^b^	23% (24/104)^b^	ND	ND	ND	ND	ND
Takemoto et al. (2009)	-	125	Retrospective	ND	57% (71/125)^b^	ND	ND	ND	ND	ND	ND
**Shukla et al. (present study)**	**30**	**70**	**Prospective**	**25% (5/20)^a^** **71% (30/50)^b^**	**20%** **(4/20)^a^** **56% (28/50)^b^**	**10%** **(2/20)^a^** **40% (20/50)^b^**	**40%** **(12/30)^a^** **71%** **(50/70)^b^**	**33% (10/30) ^a^** **57% (40/70) ^b^**	**33% (10/30) ^a^** **45% (32/70)^b^**	**Yes**	**Yes**

^a^Precancer tissues, ^b^Cancer tissues; ND - not determined.

Appearance of activated STAT3 in early precancers and especially those with HPV16 infection and its specific localization in basal and suprabasal cells (Figure 4) which represent the proliferating compartment of an epithelium in which transformation process takes place and is the site of productive HPV infection, clearly reflects a possible involvement of STAT3 in establishment of persistent viral infection in HPV infected lesions. Interestingly, recent study identifying cancer stem-like cells from primary cervical carcinoma also demonstrated high expression of STAT3 mRNA in all of the sphere-forming stem cells [34]. Not surprising, constitutive STAT3 expression and its activation, to some extent, were also observed in some of the normal tissue with inflammatory cytology (Figure 3C lane N2). It is likely that, inflammation caused by primary infection with sexually transmitted pathogens like *Chlamydia trachomatis*, may enhance STAT3 activation which may further support HPV infection and persistence. Sexually transmitted infections like *Neisseria gonorrhea*, *Chlamydia trachomatis*, and Herpes Simplex virus 2 have also been shown to act as cofactors for cervical cancer development [35-37] and are often associated with an intense chronic inflammatory response and microulcerations in the cervical epithelium [38] that cause exposure of the basal cell layer of the epithelium to infectious HPV virions and subsequent viral entry.

Present study demonstrated a significant correlation between HPV16 positivity and overexpression of STAT3 and pSTAT3 in cervical precancer and cancer lesions. Interestingly, some of the HPV16^+ ^precancers and cancer cases particularly the lesions with WDSCC histopathology showed a lower level of STAT3/pSTAT3 expression and nuclear positivity. Although HPV16 positivity among various histopathological grades (WDSCC, MDSCC, PDSCC) differed only marginally (86%, 80%, 80% respectively) it did not similarly correlate with STAT3 expression or nuclear localization. Histopathologically more advanced HPV16^+ ^PDSCC cases expressed higher levels of nuclear localized STAT3 in comparison to HPV16^+ ^WDSCC cases. This reflects that mere presence of viral DNA in host cells does not induce STAT3 activity but requires expression of viral genes/oncogenes to interact with host cell signaling that governs activation of STAT3 signaling cascade and the absence of these factors may result in diminished STAT3 activity. Moreover, physical state of viral DNA (integrated versus episomal) and the copy number of the virus that influence the magnitude of viral oncogene expression [39,40] could be the important factors responsible for variable cellular response with respect to levels of STAT3 activity. However, these upstream mechanisms are yet to be investigated.

Constitutively active STAT3 has been shown to be associated with higher histological grades and invasive cancer in several epithelial and other malignancies [12,18]. Although the reasons for aberrant STAT3 activity in cervical precancer and cancer lesions remains to be investigated, its association with HPV16 infection in cervical carcinogenesis is evident from data presented in the present study. Studies indicate high viral oncogenes E6/E7 mRNA expression or increased viral genomes/cell strongly relate to advanced histopathological grades that favor poor prognosis [41,42]. Similarly, STAT3 has also been shown to be a poor prognostic factor in cervical cancer by other investigators [20]. The relation between increased STAT3 expression with HPV16 copy number or its oncogene expression is currently not known, though several lines of evidence suggest a possible interaction between these two regulatory arms of cervical carcinogenesis. Similar to HPV E7 induced transformation in cervical cells by targeting retinoblastoma (Rb) activity [43], Simian Virus 40-induced transformation caused by inactivation of pRB through its large T antigen has been shown to result in upregulation of STAT3 [44]. On the other hand, p53 and STAT3 have been shown to antagonize expression of each other as p53 prevents the effect of STAT3 on cell transformation [45] and STAT3 down-regulates the expression and function of p53 by binding to the p53 promoter and resulting in decreased *de novo *expression of p53 [46]. These observations indicate that aberrantly expressed STAT3 that drive oncogenic transformation could be a potential outcome of E6/E7-mediated de-stabilization of p53/pRB-mediated cell cycle regulatory loop that keeps negative control over STAT3 expression. Indeed, our recent observations do indicate an increased STAT3 mRNA expression in cervical cancers [47] which was also validated in the current study. Current findings demonstrate that these transcriptionally-overexpressed STAT3 transcripts are significantly translated as functional proteins which are simultaneously activated through phosphorylation events and may be playing an important role in driving HPV16 mediated cervical carcinogenesis.

We observed a constitutively active STAT3 in cervical cancer cases which increased as a function of disease severity. STAT3 activity is regulated by two independent phosphorylation, at Tyr705 and at Ser727 which are required for its fully functional activity [14]. Tyr705 phosphorylation primarily controlled by STAT3 upstream kinases, JAK, Src and EGFR or its negative regulators like phosphatases PTEN, SOCS and PIAS, whereas Ser727 phosphorylation is regulated by MAP/JNK pathway [48] that is commonly activated in stress response and chronic inflammation. However, there is no report that directly or indirectly demonstrates the interaction of HPV oncogenes with these positive or negative regulators of STAT3 activity. Since chronic inflammation is the precursor of majority of cancers [49], a likely-hood of increased expression of various inflammatory cytokines due to HPV infection cannot be ignored in cervical carcinogenesis. Inflammatory cytokine IL-6, a potent inducer of STAT3 activity through binding to gp130 associated receptors and Jak/Tyk kinases, has received particular attention in the pathogenesis of cervical cancer. Non-malignant HPV-transformed keratinocytes and cervical carcinoma cells produce large quantities of IL-6 [50]. However, studies show that IL-6 may not be working in the autocrine manner as the cervical cells tend to lose the IL-6 receptor [50]. Therefore, constitutive activation of STAT3 could be an IL-6-independent event promoted by alternate signaling pathway. Studies indicate high expression of epidermal growth factor receptor (EGFR) RNA in three dimensional organotypic cultures of human cervical carcinoma cells and blocking EGFR functions by a specific and reversible inhibitor, PD153035, decrease the DNA synthesis and inhibited invasion in a dose-dependent manner [51]. EGFR has been shown to initiate multistage skin carcinogenesis in murine models through activation of STAT3 [52] and ErbB2 and EGFR family receptors have been demonstrated to be frequently amplified in squamous cell carcinoma of uterine cervix [53,54]. An increased STAT3 activation observed in HPV-infected cells could be due to constitutive activation of EGFR [55]. Alternatively, epigenetic alternations of expression of negative regulators like PTEN, SOCS3 or PIAS could be another possible reason for aberrantly activated STAT3. In low risk HPV6/11-associated laryngeal papillomas, presence of a moderate or low level pSTAT3(Tyr705) was detected in non-anogenital papillomas which was attributed to the increased expression of its negative regulator, PTEN [56]. Interestingly, PTEN methylation and loss of PTEN expression are early events in the development of cervical cancer [57]. Our preliminary investigations looking into the promoter methylation of SOCS and PIAS gene has revealed significantly higher methylated promoter in cervical precancer and cancer lesions (unpublished observation) which is being investigated further.

Overall present study demonstrates an aberrant expression and constitutive activation of STAT3 in cervical carcinogenesis that accumulates gradually during the process of cervical carcinogenesis and describes a significant correlation of high risk HPV16 infection in cervical lesions with active STAT3 expression. However, the interaction of HPV16 or its oncogenes with STAT3 signaling in cervical cancer and the mechanism of HPV16-mediated STAT3 activation is yet to be elucidated. Understanding mechanism of disease pathogenesis particularly focusing on interaction of HPV genes/oncogenes with STAT3 signaling may help in development of novel approaches for therapeutic interventions against HPV infection and cervical cancer.

## Abbreviations

EMSA: electrophoretic mobility shift assay; EGFR: epidermal growth factor receptor; HPV: human papillomavirus; HSIL: high grade squamous intraepithelial lesion; LCR: long control region; LSIL: low grade squamous intraepithelial lesion; PBS: Phosphate buffer saline; STAT3: Signal transducer and activator of transcription 3; VEGF: vascular endothelial growth factor

## Competing interests

The authors declare that they have no competing interests.

## Authors' contributions

SS participated in the design and interpretation of the study, carried out the majority of the experimental work and helped to draft the manuscript. GS, SH and AP assisted with sample collection, HPV diagnosis, and Immunohistochemistry. SM assisted with EMSA, western blot and tissue culture and participated in the design and interpretation of the study and helped to draft the manuscript. S Batra and S Bhambhani performed clinical evaluation, staging, histopathological grading and tumor diagnosis. SFB participated in the design and interpretation of the study and helped to draft the manuscript. BCD participated in the design and interpretation of the study and helped to draft the manuscript. ACB, conceived and designed the study, participated in the interpretation of the data and critically reviewed and communicated the manuscript. All authors have read and approved the final manuscript.

## Supplementary Material

Additional file 1 **Table S1**. Expression of STAT3 and phosphorylated STAT3 [pSTAT3(Y705) and pSTAT3(S727)] in normal, pre-cancer and cancer lesions of the uterine cervix as observed by immunoblotting. Click here for file

Additional file 2 **Table S2**. Immunohistochemical analysis of STAT3 and phosphorylated STAT3 [pSTAT3 (Y705) and pSTAT3 (S727)] expression in normal, pre-cancer and cancer lesions of the uterine cervix. Click here for file
