# Supplementary material for: Aberrant expression and constitutive activation of STAT3 in cervical carcinogenesis: implications in high-risk human papillomavirus infection
Source: Mol Cancer. 2010 Oct 27;9:282. doi: 10.1186/1476-4598-9-282 (PMC2984472; doi:10.1186/1476-4598-9-282)
Supplement: Additional file 1 — Table S1. Expression of STAT3 and phosphorylated STAT3 [pSTAT3(Y705) and pSTAT3(S727)] in normal, pre-cancer and cancer lesions of the uterine cervix as observed by immunoblotting. [file 1476-4598-9-282-S1.DOC]

**Additional File 1(Supplementary Table S1): Expression of STAT3 and phosphorylated STAT3 [pSTAT3(Y705) and pSTAT3(S727)] in normal, pre-cancer and cancer lesions of the uterine cervix as observed by immunoblotting^1^**

| **Target Protein** | **Normal (n=20)** | | | | **Pre-cancer**  **(n=30)** | | | | **Cancer (n=70)** | | | | ***p* value** |
| --- | --- | --- | --- | --- | --- | --- | --- | --- | --- | --- | --- | --- | --- |
| **Expression**  **Level (→)** | **Nil (-)** | **Weak (+)** | **Medium**  **(++)** | **Strong (+++)** | **Nil (-)** | **Weak (+)** | **Medium**  **(++)** | **Strong (+++)** | **Nil (-)** | **Weak (+)** | **Medium**  **(++)** | **Strong (+++)** |  |
| **STAT3** | 14 | 4 | 1 | 1 | 7 | 11 | 6 | 6 | 6 | 14 | 13 | 37 | **0.04^a^**  **0.000003^b^**  **0.006^c^** |
| **pSTAT3 (Y705)** | 16 | 3 | 0 | 1 | 10 | 10 | 4 | 6 | 9 | 21 | 7 | 33 | **0.04^a^**  **0.0001^b^**  **0.05^c^** |
| **pSTAT3**  **(S727)** | 16 | 3 | 0 | 1 | 16 | 4 | 6 | 4 | 27 | 13 | 13 | 19 | **0.04^a^**  **0.004^b^**  0.5^c^ |

^1^Arbitrary level of expression in immunoblotting: Strong = (++++); Medium = (++); Weak = (+); Nil / not detectable = (-). Values indicate the distribution of specimens in each category.

*p* value, probability from Fischer’s Exact Test comparing the expression of proteins (Nil + Low versus Moderate + Strong) among: ^a^pre-cancer versus controls; ^b^cancer versus controls and ^c^cancer versus pre-cancer. Bold type refers to statistically significant *p* values.
