# Supplementary material for: Aberrant expression and constitutive activation of STAT3 in cervical carcinogenesis: implications in high-risk human papillomavirus infection
Source: Mol Cancer. 2010 Oct 27;9:282. doi: 10.1186/1476-4598-9-282 (PMC2984472; doi:10.1186/1476-4598-9-282)
Supplement: Additional file 2 — Table S2. Immunohistochemical analysis of STAT3 and phosphorylated STAT3 [pSTAT3 (Y705) and pSTAT3 (S727)] expression in normal, pre-cancer and cancer lesions of the uterine cervix. [file 1476-4598-9-282-S2.DOC]

**Additional File 2 (Supplementary** **Table S2): Immunohistochemical analysis^1^ of STAT3 and phosphorylated STAT3 [pSTAT3 (Y705) and pSTAT3 (S727)] expression in normal, pre-cancer and cancer lesions of the uterine cervix**

| **Target protein** | **Staining intensity** | **Normal**  **(n=20)** | **Pre-cancer**  **(n=20)** | **Invasive Cancer (n=50)** | ***p* value** |
| --- | --- | --- | --- | --- | --- |
| **STAT3** | Nil (-) | 15 | 8 | 8 | 0.4^a^  **0.005^b^**  **0.0004^c^** |
|  | Weak (+) | 3 | 7 | 12 |
|  | Moderate (++) | 1 | 3 | 10 |
|  | Strong (+++) | 1 | 2 | 20 |
| **pSTAT3 (Y705)** | Nil (-) | 17 | 9 | 8 | 0.3^a^  **0.0002^b^**  **0.01^c^** |
|  | Weak (+) | 2 | 7 | 14 |
|  | Moderate (++) | 1 | 2 | 08 |
|  | Strong (+++) | 0 | 2 | 20 |
| **pSTAT3(S727)** | Nil (-) | 15 | 9 | 12 | 0.4^a^  **0.03^b^**  **0.002^c^** |
|  | Weak (+) | 5 | 9 | 18 |
|  | Moderate (++) | 0 | 1 | 08 |
|  | Strong (+++) | 0 | 1 | 12 |

^1^Arbitrary staining intensity grades of respective proteins in immunohistochemistry: Strong = (++++); Medium = (++); Weak = (+); Nil / not detectable = (-). Values indicate the distribution of specimens in each category.

*p* value, probability from Fischer’s Exact Test comparing the expression of proteins (Nil + Low versus Moderate + Strong) among: ^a^pre-cancer versus controls; ^b^cancer versus controls and ^c^cancer versus pre-cancer. Bold type refers to statistically significant *p* values.
